# Diffusion differences between pilocytic astrocytomas and grade II ependymomas

**DOI:** 10.2478/v10019-011-0011-x

**Published:** 2011-03-29

**Authors:** Goran Pavlisa, Gordana Pavlisa, Marko Rados

**Affiliations:** 1 Department of Radiology, University Hospital Center Zagreb, University of Zagreb School of Medicine, Zagreb, Croatia; 2 Intensive Care Unit, Special Hospital for Pulmonary Diseases, Zagreb, Croatia

**Keywords:** pilocytic astrocytoma, ependymoma, apparent diffusion coefficient

## Abstract

**Background:**

The aim of our study was to differentiate between cerebellar pilocytic astrocytomas and grade II ependymomas on the basis of their diffusion properties.

**Patients and methods:**

The study prospectively included 12 patients with pilocytic astrocytomas and 5 with ependymomas. Apparent diffusion coefficients (ADC) were compared between tumour types.

**Results:**

ADC values were significantly higher in pilocytic astrocytomas than ependymomas, with almost no overlapping of the range of measured ADCs between the two tumour types.

**Conclusions:**

Significant diffusion differences between pilocytic astrocytomas and grade II ependymomas enable their preoperative distinction, in combination with conventional magnetic resonance images.

## Introduction

Infratentorially located pilocytic astrocytomas and grade II ependymomas may have similar magnetic resonance imaging (MRI) appearance. Pilocytic astrocytoma (PA) is a World Health Organization (WHO) grade I tumour, with the low mitotic activity and very low potential for malignant transformation.^1^ Solid portions of PA have low cell density with bipolar „piloid“ astrocytes and characteristic microcytic component and Rosenthal fibers^2^, which represents an environment with a relatively unrestricted diffusion of water molecules in extracellular space. One of the common locations of PAs in children and young adults is cerebellum, near the fourth ventricle, which is also a predilection site for ependymomas (EPN). EPNs usually have a higher cell density compared to PAs, and according to their histological characteristics they may be classified as WHO grades I – III.^1^,^3^ Although these two tumour types are readily differentiated on the basis of MRI morphology in a majority of patients, in cases of a solid PA without typical cystic component, differential diagnosis may be difficult.^4^ Diffusion properties of intracranial tumours have been extensively studied, however, with disparate results.

The aim of our study was to differentiate between cerebellar pilocytic astrocytomas and grade II ependimomas on the basis of their diffusion properties. We hypothesised that pilocytic astrocytomas have higher values of apparent diffusion coefficient (ADC) than more cellular ependymomas.

## Patients and methods

The study prospectively included 17 patients; 12 with newly discovered and subsequently histopathologicaly proven pilocytic astrocytomas (WHO grade I), and 5 with ependymomas (WHO grade II). The neuropathologist was blinded to the MRI findings and ADC values. The adequate study power was calculated based on the pilot study, which included 8 patients, four with PAs and four with EPNs. The required sample size was 5 patients with PAs and EPNs, respectively, based on a level of reliability 1-α ≥ 0.95 and statistical power 1-β ≥ 0.8. We excluded patients with tumours smaller than 1 cm in largest diameter, because such size did not enable precise ADC measurements and the avoidance of partial volume effect. We also excluded patients with extensive artefacts of diffusion-weighted images (DWI) and those with other pathological processes besides tumour, which did not allow for the precise measurement of the control ADC sample.

MR imaging was performed on a 1.5-T system (Symphony, Siemens Medical Systems, Erlangen, Germany) with 30-mT/m gradients and a slew rate of 125 T/m/s. Single-shot echo-planar DW images were acquired in a transverse plain with the acquisition of a diffusion trace, with the following parameters: FOV, 22.8×22.8 cm; matrix, 128×128; slice thickness, 5 mm; slice gap, 1.5 mm; three *b* values (0, 500, and 1000 s/mm^2^); TR, 3200 ms; TE, 94 ms; Nex, 1; TA, 1 min 12 s. ADC maps were automatically calculated, according to the following equation: ADC=ln (*S*_0_/*S*_1_)/(*b*_1_−*b*_0_)×10^−5^ mm^2^/s.^5^ DWI was performed before the administration of gadolinium-DTPA in all cases. The imaging protocol also included conventional sequences: in all cases, axial FSE T2WI and axial nonenhanced and contrast-enhanced SE T1WI. Two neuroradiologists separately defined the following areas on conventional images, with a consensus in cases of disagreement:

1. solid tumour, as an area with a mass effect and contrast enhancement;
2. normal brain tissue, as an area with normal signal intensities in all sequences, without mass effect;
3. cystic/necrotic area, as an area with a hypointense signal in T1WI and a hyperintense signal in T2WI, without contrast enhancement;
4. haemorrhage, as an area with a hyperintensity in nonenhanced T1WI; and
5. calcified tumours, as hypointense areas in DWI b=0 images.

Cystic/necrotic areas and areas containing haemorrhage and calcifications were excluded from further analysis. ADC measurements were performed using a region-of-interest (ROI) method, with uniform ellipsoid ROIs of 0.2 cm^2^, containing approximately 10 pixels. ADC measurements were performed using e-Film Workstation 2.1 (Merge Healthcare, Milwaukee, WI, USA), with a simultaneous display of contrast-enhanced T1WI, T2WI, isotropic DWI, and ADC map. We placed three ROIs in the areas corresponding to each tumour. The representative value used in data and statistical analysis was the mean value±S.D. One control ROI was placed in normal tissue. We additionally preformed ADC measurements bilaterally in deep white matter to exclude any laterality differences of the healthy tissue. The mean tumour ADC values±S.D. were compared to normal tissue, and between PAs and EPNs. The comparison of differences in mean ADC values was performed using the Mann–Whitney *U* test. *P*<0.05 was considered statistically significant. A statistical analysis was performed using StatView software (SAS Institute Inc. Version 5.0.1) and Statistica 7 (StatSoft, Inc., Netherlands).

All the patients gave their informed consent, and the study was approved by the institutional review board.

## Results

There were no significant differences of ADC of normal deep white matter between brain hemispheres in patients with both investigated tumour types; it was 80 − 85 × 10^−5^ mm^2^/s. Continuous variables are described in Table 1.

The average age was 19 in patients with PAs, and 20 years in patients with EPNs.

The difference of ADCs between tumour and normal brain tissue of patients with PAs was statistically highly significant. In patients with EPNs, there was no significant difference of ADC between tumour and normal tissue. ADC was significantly higher in PAs compared to EPNs (Table 2). The mean ADC of PAs was 156.7 × 10-5 mm^2^/s, while the mean ADC of EPNs was 97.6.

**FIGURE 1.**
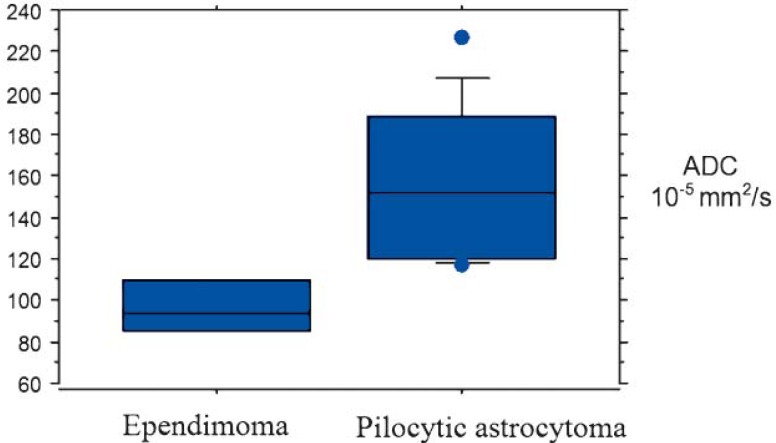
The range of apparent diffusion coefficient (ADC) values of ependymo-mas and pilocytic astrocytomas. ADCs of these tumours were minimally overlapping, between values of 117.5 × 10^−5^ mm^2^/s and 121.9 × 10^−5^ mm^2^/s.

The range of ADCs of investigated tumours is displayed graphically (Figure 1).

## Discussion

**FIGURE 2.**
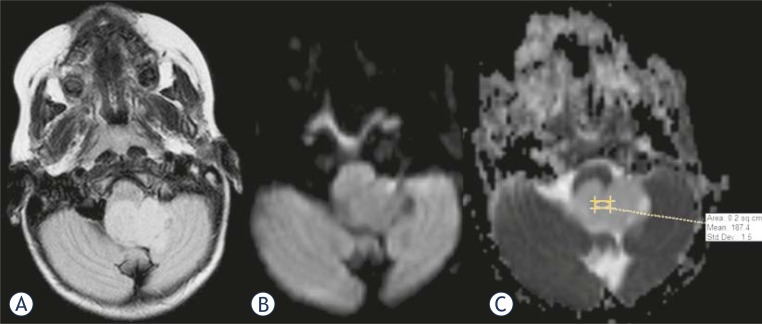
MR images in axial plane of a patient with pilocytic astrocytoma. A: Fluid-attenuated inversion recovery (FLAIR) image with a homogenous, slightly hyperintense tumour in the fourth ventricle and left foramen of Luschka. Diffusion-weighted image (B), and map of apparent diffusion coefficient (ADC) (C) without restriction of the diffusion. Intratumoural diffusion coefficient is 187.4 × 10^−5^ mm^2^/s.

**FIGURE 3.**
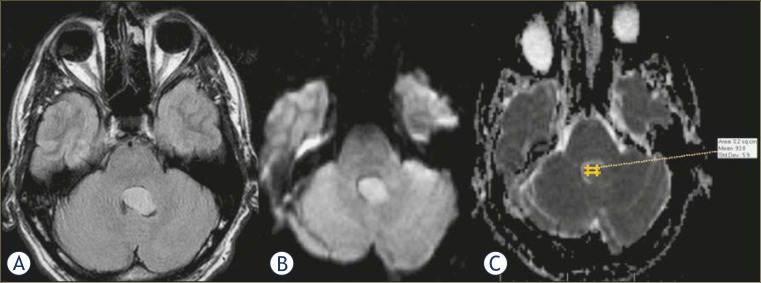
Axial MRI of a patient with grade II ependymoma shows morphologically similar tumour to the one seen in Figure 1A, with moderate hyperintensity in FLAIR image. Diffusion-weighted image and ADC map reveal relatively restricted diffusion, with coefficient of 93.8 × 10^−5^ mm^2^/s.

Pilocytic astrocytoma and ependymoma, together with medulloblastoma, are the most common cerebellar tumours in children and young adults.^6^ Since these tumours have different biologic potential, the preoperative differentiation among them has important consequences on treatment planning. Conventional MRI, although being the most important diagnostic tool in brain imaging, is not sufficiently specific for the differentiation of these tumour types in all patients. Pilocytic astrocytomas are typically characterized by strongly contrast-enhancing *nodus* and the variably large cystic component, filled with rare proteinaceous fluid which resembles the signal from cerebrospinal fluid. They are usually well-delineated from the surrounding brain tissue, and located in posterior fossa. Ependymomas often share the same location with pilocytic astrocytomas in pediatric patients, but are mostly solid tumours, typically with ependymal spread through the fourth ventricle and into ventricular foramina. However, both tumour types may have morphologically very similar appearance, since not all pilocytic astrocytomas have a pronounced cystic component (Figure 2,3).

Therefore, we aimed to differentiate between PAs and EPNs by measuring intratumoural apparent diffusion coefficient. This method is currently available on virtually all MRI scanners; it is non-invasive, with short acquisition times and is free from motion artefacts compared to other imaging sequences. It is widely used in diagnostics of stroke, as well as other pathological conditions.^7^,^8^ Differentiation of intracranial tumours on the basis of their ADC values has been relatively extensively studied, however, with disparate results.^9^–^16^

We investigated ADC differences in 12 patients with pilocytic astrocytomas and 5 patients with ependymomas. We did not include patients with medulloblastomas in the study, since the literature results on diffusion in highly cellular medulloblastomas are rather clear, with signs of restricted diffusion in previous studies.^14^–^17^ Ependymomas included in the study were WHO grade II. Tumours of grade I, myxopapillary ependymoma and subependymoma, were excluded since they have distinct morphological, biological and demographic features, the first presenting almost exclusively in *cauda equina* region, and the second in an older age group and with different MRI characteristics than ependymoma grade II.^18^ PAs have low cell density with relatively large volume of extracellular space, unlike EPNs, therefore, we hypothesised that the diffusion of water molecules in PAs is of a higher order compared to EPNs. Our results confirmed that assumption, with significantly higher ADC values in PAs than EPNs and with almost no overlapping of the range of measured ADCs between the two tumour types. The intratumoural ADC values of our patients were in line with some previous investigations^15^, while others found higher ADC in EPNs^14^, or lower ADC in PAs^19^, compared to our patients. The ADC differences among these studies may be due to the retrospective character of previous studies, different designs without a direct comparison of these tumour types, a single measurement in each tumour, thicker DWI slice used in imaging or due to the investigation of exclusively paediatric population.

Apparent diffusion coefficients of pilocytic astrocytomas and ependymomas in our patients were reliable indicators of tumour type. The level of ADC above 120 × 10^−5^ mm^2^/s was indicative of PA, while the ADC between 80 and 120 × 10^−5^ mm^2^/s was characteristically for EPN. We believe that this difference of diffusion properties is due to histological features of investigated tumours. The structure of PAs is „biphasic“, consisting of vacuolated low density areas and areas of relatively higher density, however even in the latter areas, the overall cellularity is low to moderate, with small nuclei and microcytic stroma.^20^ This enables relatively unrestricted diffusion of water molecules, unlike in EPNs, which is of uniformly moderate cell density. The brain tissue which was normal on conventional MRI had very similar ADC levels in both brain hemispheres of our patients, excluding any laterality differences.

In conclusion, a significantly higher apparent diffusion coefficient in pilocytic astrocytomas compared to WHO grade II ependymomas probably reflects the differences in cell density of these tumours, and enables their preoperative distinction, in combination with conventional magnetic resonance images.

## Figures and Tables

**TABLE 1.** Description of continuous variables

**Tumour type**	**ADC (N × 10^−5^ mm^2^/s)**				

	**Mean**	**Range**	**SD**	**VC**	**SE**
**Pilocytic astrocytoma**	156.7	117.5 – 226.9	38	0.244	11
**Ependymoma**	97.6	80.4 – 121.9	17	0.181	8

ADC = apparent diffusion coefficient, SD = standard deviation, VC = variability coefficient, SE = standard error

**TABLE 2.** Differences of tumour ADC in patients with pilocytic astrocytomas and ependymomas

**Tumor type**	**ADC (N × 10^−5^ mm^2^/s) ± SD**		**p**

	**Tumor**	**Normal tissue**	
**Pilocytic astrocytoma**	156.7 ± 38	85.7 ± 9	<0,0001

**Ependimoma**	97.6 ± 17	83.8 ± 12	0.3858

**p**	0.0212	0.9035	

ADC = apparent diffusion coefficient, Underlined = statistically significant difference.
